# Restrictive Potassium Supplementation Strategy in a General Intensive Care Unit: A Before-and-After Quality Improvement Audit in a Mixed Critical Care Population

**DOI:** 10.7759/cureus.118434

**Published:** 2026-10-23

**Authors:** Deveshwar Bhalla, Rasi Mizori, Waqas Akhtar, Riaz Aziz, Hementi Patel, Fraser Hanks, Andrew Retter, Angelo Sousa, Diogo Braga

**Affiliations:** 1 Intensive Care Unit, Guy's and St Thomas' NHS Foundation Trust, London, GBR; 2 Critical Care Medicine, King's College London, London, GBR; 3 Critical Care Medicine, Guy's and St Thomas' NHS Foundation Trust, London, GBR; 4 Pharmacy, Guy's and St Thomas' NHS Foundation Trust, London, GBR; 5 Research, Guy's and St Thomas' NHS Foundation Trust, London, GBR

**Keywords:** atrial fibrillation (af), general critical care, intensive care unit, potassium, quality improvement research

## Abstract

Purpose: Potassium supplementation thresholds in intensive care vary widely and have historically lacked strong supporting evidence. The TIGHT K trial demonstrated non-inferiority of a restrictive potassium threshold in cardiac surgical patients; applicability to general intensive care populations is unclear.

Methods: Retrospective before-and-after quality improvement audit in a mixed adult intensive care unit (ICU), comparing two one-week periods before (May 2025) and after (September 2025) reducing the intravenous potassium supplementation threshold from <4.5 to <3.6 mmol/L.

Results: Forty-eight patients were included (22 pre-, 26 post-guideline) with comparable baseline characteristics. Mean intravenous potassium administrations per patient fell by 17.4% (from 9.41 to 7.77 administrations per patient), and the proportion of supplementation episodes initiated at K⁺ ≥4.0 mmol/L fell from 32.9% to 11.4% (a 21.5 percentage-point absolute reduction, equivalent to a 65.2% relative reduction). Pre-supplementation potassium was lower post guideline (3.44 vs 3.83 mmol/L; p <0.001), but potassium correction (ΔK) was unchanged (p = 0.51), and arrhythmia burden did not increase.

Conclusion: A restrictive potassium supplementation threshold reduced potassium use without evidence of harm in a general intensive care population, supporting wider applicability beyond cardiac surgery, although larger prospective studies are needed.

## Introduction

Management of electrolyte disturbances is a fundamental aspect of comprehensive care in intensive care units (ICUs) worldwide. Potassium is central to cardiac electrophysiology, particularly in determining the resting membrane potential and facilitating repolarisation. Hypokalaemia increases myocardial excitability and has been linked to both atrial and ventricular arrhythmias [1]. For this reason, many centres routinely supplement potassium to maintain normal levels [2-4]. However, the target range and practice vary widely between institutions and have historically lacked strong evidence to support their effectiveness [5,6]. Furthermore, frequent intravenous potassium supplementation carries increased economic and environmental cost, nursing workload, and potential patient risk [2,7].

In cardiac surgery, postoperative atrial fibrillation following cardiac surgery is the most frequent complication post coronary artery bypass graft (CABG) surgery, occurring in approximately 30% of patients, with around 90% of cases developing within the first five postoperative days [8]. This is associated with increased morbidity, prolonged critical care and hospital length of stay, higher rates of stroke, and increased healthcare resource use [9]. In the TIGHT K trial, a large multicentre non-inferiority study involving patients undergoing isolated CABG, a lower supplementation threshold of <3.6 mmol/L was found to be non-inferior to a high-normal threshold of <4.5 mmol/L in preventing new-onset atrial fibrillation [3]. Importantly, the relaxed supplementation strategy did not increase ventricular arrhythmias or adverse outcomes, and resulted in a substantial reduction in potassium use and associated cost.

The key question is whether these findings are more broadly applicable to the general intensive care population, with limited observational data previously published [7]. The TIGHT K trial demonstrated that, even in a cohort of patients theoretically at high risk from dyskalaemia-related dysrhythmias, maintaining higher potassium targets in the intensive care setting conferred no clear clinical benefit. This challenges the rationale for routine potassium supplementation above physiological norms in critically ill patients more generally. Given that intravenous potassium replacement is an invasive intervention associated with potential harms, including arrhythmogenesis, infusion-related complications, and resource burden, its liberal use warrants re-evaluation. We assessed the impact of a change in routine supplementation practice within a general ICU in the United Kingdom.

The aim of this quality improvement audit was to evaluate the effect of reducing the intravenous potassium supplementation threshold from <4.5 to <3.6 mmol/L in a general adult ICU. The primary outcomes were the number of intravenous potassium administrations, pre- and post-supplementation serum potassium, and cardiac arrhythmia. The secondary outcomes were the change in serum potassium following supplementation, hyperkalaemic overshoot, appropriateness of supplementation against the operative threshold, and drug acquisition cost.

## Materials and methods

Study design and setting

This was a retrospective review conducted in a mixed adult ICU at a tertiary academic teaching hospital (Guy's and St Thomas' NHS Foundation Trust, London, England). All patient data were extracted from the Epic electronic health record system (Epic Systems Corporation, Verona, WI, USA). Ethical approval was not required, and this was registered as a service evaluation and quality improvement project.

Intervention

The historical, or pre-guideline, IV potassium supplementation threshold was serum potassium <4.5 mmol/L. Following review of the TIGHT K randomised controlled trial [3], a revised institutional threshold of <3.6 mmol/L was implemented in July 2025. Potassium chloride was administered intravenously via a central venous catheter, and no potassium was given peripherally. The standard preparation was 20 mmol in 50 mL of 0.9% sodium chloride (362 of 409 episodes, 88.5%); the remainder were premixed 10 to 40 mmol infusion bags. The revised threshold was an institutional guideline rather than a mandatory rule. Clinicians could use different, including higher, potassium targets based on individual clinical judgement, for example in patients at high risk of ventricular arrhythmia, in line with recent evidence from the POTCAST trial [10].

Audit periods

Data were collected across two one-week audit periods: a pre-guideline period (May 2025) and a post-guideline period (September 2025). Each period was selected to represent routine clinical activity without known confounding service changes.

Study population

All adult patients (aged ≥18 years) admitted to the ICU during each audit period were included. Patients were identified retrospectively via EPIC. Inclusion and exclusion criteria are summarised in Table 1.

**Table 1:** Patient inclusion and exclusion criteria.

Inclusion criteria	Exclusion criteria
Adult patients aged ≥18 years	Age <18 years
Admitted to the mixed adult ICU during an audit period	ICU admission outside the specified audit periods
Audit periods: May 2025 (pre-guideline) or September 2025 (post-guideline)	No intravenous potassium supplementation administered during the ICU stay
Blood gas serum potassium value available both immediately before and after supplementation	

Data collection

For each patient, the following data were recorded: age, sex, primary diagnosis, admission specialty, cardiac history, and IV potassium supplementation episodes. For each episode (the entire intensive care stay), pre-supplementation and post-supplementation serum potassium concentrations on blood gas results, dose administered, route of measurement (arterial blood gas or venous blood gas), and cardiac rhythm at the time of supplementation were extracted. Admission specialty was classified as cardiac (including cardiac medical admissions, cardiac surgery, and cardiac arrest) or non-cardiac (including pneumonia, post renal transplant, diabetic ketoacidosis and sepsis) based on the primary reason for ICU admission.

Appropriateness of supplementation was defined as an episode in which intravenous potassium was initiated at a serum concentration below the threshold specified by the operative guideline during the relevant audit period (i.e., <4.5 mmol/L pre-guideline and <3.6 mmol/L post-guideline). Arrhythmia was defined as any documented cardiac rhythm other than normal sinus rhythm or sinus tachycardia. Cardiac rhythm was monitored continuously, with monitoring data uploaded automatically to Epic; the rhythm at each supplementation episode and rhythm duration data were extracted from these records and expressed in hours.

Outcomes

The primary outcomes were mean serum potassium concentration, number of intravenous potassium administrations, and incidence of cardiac arrhythmias. Secondary outcomes included the change in serum potassium following supplementation (ΔK = post-supplementation potassium − pre-supplementation potassium), mean pre- and post-supplementation potassium levels, incidence of hyperkalaemic overshoot (K > 5.0 mmol/L), and appropriateness of potassium replacement as defined by local guideline criteria. Cost of potassium was calculated using the drug acquisition price of a 20 mmol intravenous potassium chloride pre-filled syringe (B. Braun SE, Melsungen, Germany; £13.28 per unit), based only on product cost and excluding nursing time, preparation, or waste.

Statistical analysis

The analysis was conducted at both patient and episode level. The number of hypokalaemia episodes varied considerably between patients, with some experiencing a single episode and others experiencing more than 10 episodes during their admission. Treating each episode as an independent observation would, therefore, have resulted in individual patients contributing disproportionately to the analysis. For the patient-level analysis, the period of potassium supplementation was considered as a single treatment period. The potassium concentration immediately prior to the first episode requiring supplementation was defined as the pre-supplementation potassium, while the final potassium concentration following supplementation, after which no further potassium replacement was required, was defined as the post-supplementation potassium. The same approach was applied to the assessment of cardiac rhythm. The rhythm recorded immediately prior to the first potassium supplementation was defined as the pre-supplementation rhythm, and the final rhythm recorded following the last supplementation episode was defined as the post-supplementation rhythm. For analyses specifically examining individual supplementation or hypokalaemic events, episode-level data were used.

Continuous variables that followed a normal distribution were presented as mean ± standard deviation (SD). Median and interquartile ranges were used for variables that followed an asymmetrical distribution. Baseline demographic and clinical characteristics were compared between groups using the Mann-Whitney U test, reflecting their non-normal distribution. Pre- and post-supplementation potassium were compared using the Welch t-test. The change in potassium (ΔK) and administrations per patient were compared using the Mann-Whitney U test, and within-period changes in potassium using the Wilcoxon signed-rank test. No sample size calculation was performed, no adjustment was made for multiple comparisons, and p-values should be interpreted as exploratory. Categorical variables were compared using the chi-square test or Fisher's exact test. A p-value <0.05 was considered statistically significant. As individual patients contributed multiple supplementation episodes, data are reported primarily at the episode level; patient-level summaries are presented for utilisation outcomes.

## Results

Baseline characteristics

Forty-eight patients were included; 22 in the pre-guideline intervention and 26 in the post-guideline intervention (Table 2). Baseline characteristics were comparable across both periods. Mean age was 57.7 ± 21.1 years pre-guideline and 53.5 ± 18.5 years post guideline (Mann-Whitney U, U=334, p = 0.33). Male patients comprised 63.6% (14/22) and 50.0% (13/26) of each cohort, respectively (chi-square test with Yates’ correction, χ²=0.43, p = 0.51), and a history of cardiac disease was present in 50.0% (11/22) pre-guideline and 34.6% (9/26) post guideline (χ²=0.61, p = 0.43). The proportion of patients admitted for a primary cardiac reason (cardiac medical, cardiac surgery, or cardiac arrest) was 18.2% pre-guideline (n = 4) and 34.6% post guideline (n = 9; χ²=0.90, p = 0.34). No statistically significant differences were identified across any baseline characteristic (all p >0.05). The admission case mix was varied across both periods (Table 2), reflecting the mixed general adult ICU population.

**Table 2:** Baseline patient characteristics by audit period. Categorical comparisons use the chi-square test with Yates’ continuity correction; dashes indicate comparisons not formally tested.

Characteristic	Pre-guideline (n = 22)	Post guideline (n = 26)	Test (statistic)	p-value
Age, years (mean ± SD)	57.7 ± 21.1	53.5 ± 18.5	Mann-Whitney U (U=334)	0.33
Male sex, n (%)	14 (63.6%)	13 (50.0%)	Chi-square, Yates’ (χ²=0.43)	0.51
Cardiac history, n (%)	11 (50.0%)	9 (34.6%)	Chi-square, Yates’ (χ²=0.61)	0.43
Cardiac admission reason, n (%)	4 (18.2%)	9 (34.6%)	Chi-square, Yates’ (χ²=0.90)	0.34
ICU length of stay, days (mean ± SD)	9.1 ± 12.8	6.3 ± 5.7	Mann-Whitney U	0.89
Admission specialty, n (%)				
Surgical	9 (40.9%)	5 (19.2%)	—	—
Respiratory	4 (18.2%)	3 (11.5%)	—	—
Cardiac (medical)	4 (18.2%)	4 (15.4%)	—	—
Cardiac surgery (e.g. valve replacement, surgical management of infective endocarditis)	0 (0.0%)	3 (11.5%)	—	—
Cardiac arrest	0 (0.0%)	2 (7.7%)	—	—
Metabolic/Endocrine	1 (4.5%)	5 (19.2%)	—	—
Gastroenterology/Hepatology	2 (9.1%)	1 (3.8%)	—	—
Oncology/Haematology	0 (0.0%)	3 (11.5%)	—	—
Renal/Transplant	2 (9.1%)	0 (0.0%)	—	—

Potassium levels

Pre-supplementation potassium, analysed at the episode level, was significantly lower post guideline: 3.443 ± 0.458 mmol/L post guideline versus 3.832 ± 0.448 mmol/L pre-guideline (Welch t-test, t=8.69, p <0.001). Post-supplementation potassium, analysed at the patient level, was similar between groups: 4.02 mmol/L (SD 0.47) post guideline versus 4.13 mmol/L (SD 0.33) pre-guideline (Welch t-test, t=0.97, p = 0.34) (Table 3, Figure 1).

**Table 3:** Potassium supplementation outcomes by audit period. ΔK: change in serum potassium (post minus pre-supplementation). Supplementation initiated below the operative threshold (<4.5 mmol/L pre-guideline; <3.6 mmol/L post-guideline); not formally tested (see Discussion). Drug acquisition cost based on a unit price of £13.28 per 20 mmol intravenous potassium chloride pre-filled syringe (B. Braun SE, Melsungen, Germany; £13.28 per unit); excludes nursing time, preparation, and waste.

Measure	Pre-guideline	Post guideline	Test (statistic)	p-value
Total patients, n	22	26	—	—
Supplementation episodes, n	207	202	—	—
Change in potassium (ΔK), mmol/L (mean ± SD)	0.24 ± 0.51	0.28 ± 0.44	Mann-Whitney U (U=19825)	0.51
Supplementation at K ≥4.0 mmol/L, n (%)	68 (32.9%)	23 (11.4%)	Chi-square (χ²=27.23)	<0.001
Hyperkalaemic overshoot (K >5.0 mmol/L), n (%)	5 (2.5%)	1 (0.5%)	Chi-square (χ²=2.67)	0.102
Supplementation initiated below operative threshold, n (%)	198 (95.7%)	125 (61.9%)	—	—
Mean IV administrations per patient (median, IQR)	3.5 (2–6.75)	5.5 (2–9)	Mann-Whitney U (U=285)	0.99
Mean IV administrations per patient	9.41	7.77	—	—
Mean total IV potassium per patient, mmol	190	168	—	—
Estimated drug acquisition cost per patient, £ (mean)	124.95	103.18	—	—

**Figure 1:**
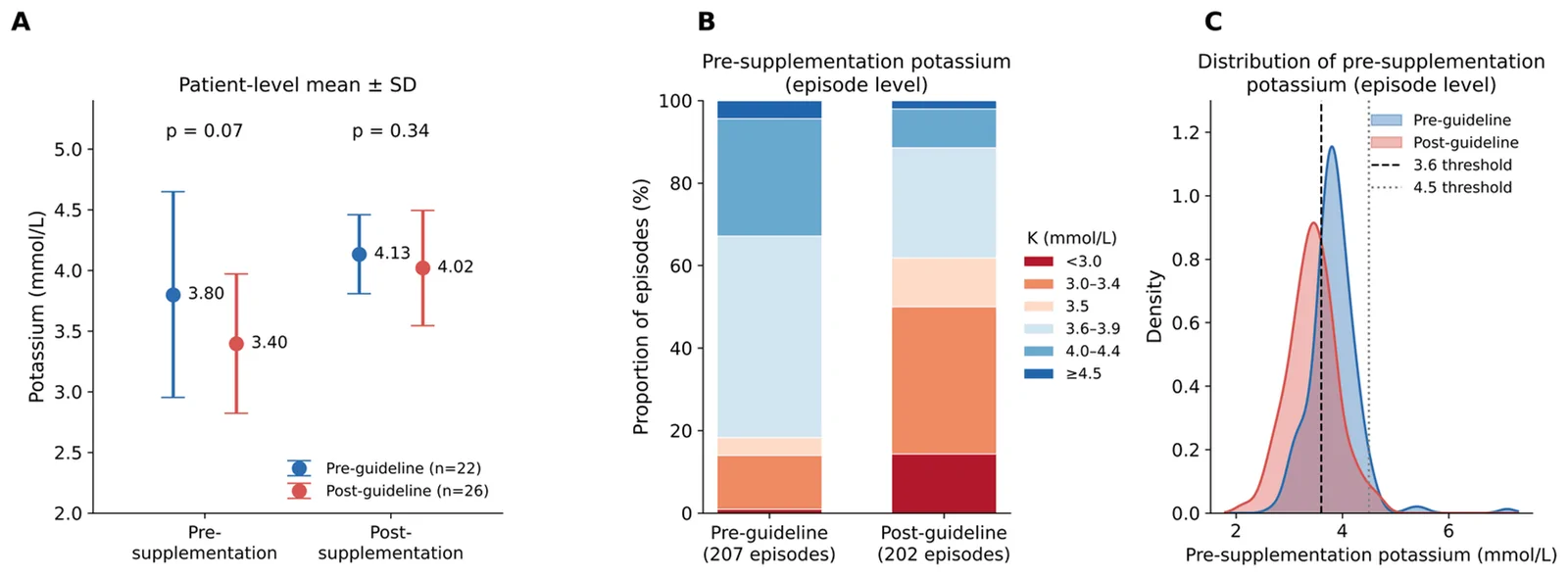
Impact of TIGHT K–aligned guideline implementation on potassium supplementation in a general ICU (A) Patient-level mean ± SD potassium before the first supplementation episode and after the last supplementation episode, by audit cycle (Welch’s t-test: pre-supplementation p = 0.07; post supplementation p = 0.34); (B) Distribution of pre-supplementation potassium categories by audit cycle (episode-level); (C) Density distribution of pre-supplementation potassium (episode-level).

Mean intravenous potassium administrations per patient fell by 17.4% (from 9.41 to 7.77; Mann-Whitney U, U=285, p = 0.99 for the median/IQR comparison), with mean total potassium per patient decreasing from 190 to 168 mmol. The median number of administrations per patient was not lower post guideline (3.5 (IQR 2 to 6.75) pre-guideline versus 5.5 (IQR 2 to 9) post-guideline), indicating that the reduction in the mean was driven by a small number of high-utilisation patients. The proportion of supplementation episodes initiated at a serum potassium ≥4.0 mmol/L fell from 32.9% (68/207) to 11.4% (23/202) (chi-square, χ²=27.23, p <0.001) (a 21.5 percentage-point absolute reduction, equivalent to a 65.2% relative reduction), indicating substantially more selective prescribing against the new threshold.

Potassium correction (ΔK) between groups was similar, with mean ΔK 0.28 ± 0.44 mmol/L post guideline versus 0.24 ± 0.51 mmol/L pre-guideline (Mann-Whitney U, U=19825, p = 0.51). Within both periods, supplementation produced a statistically significant rise in serum potassium (both p <0.001, Wilcoxon signed-rank test).

Supplementation was initiated below the operative threshold in 95.7% (198/207) of pre-guideline episodes and 61.9% (125/202) of post-guideline episodes; this comparison was not formally tested, as the appropriateness criterion does not fully account for individualised clinical decision-making (see Discussion). In 38.1% (77/202) of post-guideline episodes, supplementation was initiated at K⁺ ≥3.6 mmol/L. Hyperkalaemic overshoot (K >5.0 mmol/L) was rare in both periods (2.5% (5/204) vs 0.5% (1/202); chi-square, χ²=2.67, p = 0.102).

Cardiac rhythm analysis

Rhythm change was initially assessed at the patient level, as described in the Methods section. In the pre-implementation group, 72.7% (16/22) of patients had no change in cardiac rhythm following potassium supplementation, compared with 65.4% (17/26) in the post-implementation group. There was no statistically significant difference in the proportion of patients experiencing a change in cardiac rhythm between the two audit cycles (Fisher’s exact test, OR=1.41, p = 0.76).

Episode-level rhythm data immediately before and after supplementation are shown in Table 4. In the pre-guideline period, atrial fibrillation was the predominant arrhythmia (63 episodes before, 64 after supplementation). In the post-guideline period, atrial fibrillation was substantially less common (three and two episodes respectively), with a higher proportion of ventricular-paced rhythms (17 episodes in both windows), reflecting differences in case mix between audit periods. The proportion of episodes associated with any arrhythmia was 36.7% and 38.2% (before and after supplementation) in the pre-guideline period, compared with 14.9% and 16.8% post guideline. No consistent increase in arrhythmia prevalence was observed following supplementation in either period.

**Table 4:** Cardiac rhythm before and after potassium supplementation, by audit period. Data are presented as the number of episodes (n), except the final row (Any arrhythmia), which is presented as n (%). Arrhythmia is defined as any rhythm other than NSR or ST. Differences in atrial fibrillation prevalence between periods are attributable to case-mix variation. NSR: normal sinus rhythm; ST: sinus tachycardia; N/A: not available

Rhythm	Pre-guideline: before	Pre-guideline: after	Post guideline: before	Post guideline: after
NSR	104	100	148	144
Sinus tachycardia	23	25	24	24
Sinus bradycardia	12	14	10	15
Atrial fibrillation	63	64	3	2
Ventricular paced	1	1	17	17
N/A	4	3	0	0
Any arrhythmia (excluding NSR/ST)	76 (36.7%)	79 (38.2%)	30 (14.9%)	34 (16.8%)

Rhythm hours

Total arrhythmia hours accumulated during each audit period are shown in Table 5. Pre-guideline total arrhythmia hours were 1748.6 versus 603.0 post guideline. This was predominantly driven by AF (1301.5 vs 25.0 hours), which reflects the substantially higher starting AF burden in the pre-guideline cohort. Post-guideline ventricular-paced hours increased (441.7 vs 0.0 hours), consistent with a higher proportion of patients with implanted cardiac devices (two patients post guideline vs 0 patients pre-guideline). The number of patients experiencing any arrhythmia was 11 pre-guideline and eight post guideline.

**Table 5:** Cumulative cardiac rhythm hours by audit period. Data are presented as cumulative hours summed across all episodes in each audit period, except the final row (Patients with any arrhythmia), which is presented as n. Differences in atrial fibrillation and ventricular-paced hours between periods predominantly reflect variation in case mix rather than a treatment effect of the guideline change. NSR: normal sinus rhythm; ST: sinus tachycardia

Rhythm	Pre-guideline, hours	Post guideline, hours	Difference, hours
NSR	2127.2	2632.1	504.8
Sinus tachycardia	948.2	705.8	−242.4
Combined NSR/ST	3075.4	3337.9	262.5
Sinus bradycardia	447.1	135.1	−312.0
Atrial fibrillation	1301.5	25	−1276.5
Ventricular paced	0	441.7	441.7
Total arrhythmia hours	1748.6	603	−1145.6
Patients with any arrhythmia, n	11	8	−3

## Discussion

This single-centre quality improvement project evaluated the impact of adopting a TIGHT K-aligned restrictive IV potassium supplementation threshold (<3.6 mmol/L) in a general mixed adult ICU. The key findings were: (1) no significant change on overall patient potassium readings over the length of stay; (2) a 17.4% reduction in mean IV potassium administrations per patient (9.41 to 7.77), accompanied by a reduction in the proportion of supplementation initiated at K⁺ ≥4.0 mmol/L from 32.9% to 11.4% (a 21.5 percentage-point absolute reduction, equivalent to a 65.2% relative reduction) and an associated reduction in drug acquisition cost per patient (although, as discussed below, this may partly reflect shorter ICU stays after the change); (3) similar biochemical potassium correction (ΔK) with negligible hyperkalaemic overshoot; and (4) no evidence of increased arrhythmia burden attributable to the lower supplementation threshold.

Though the number of patients included in this study was small, no statistically significant differences were identified in age, sex, cardiac history, or admission reason (all p >0.05). The proportions of cardiac admissions were 18.2% pre-guideline and 34.6% post-guideline (p = 0.34). Only one patient in the post-guideline group met the TIGHT K study criteria. The study population was representative of the heterogeneous case mix encountered in a general adult ICU, encompassing surgical, respiratory, metabolic, oncological, and cardiac presentations.

Applying a unit acquisition cost of £13.28 per 20 mmol intravenous potassium chloride pre-filled syringe (B. Braun), the reduction in mean administrations per patient (9.41 to 7.77) corresponded to an estimated drug acquisition cost of £124.95 per patient pre-guideline versus £103.18 per patient post guideline, a reduction of £21.78 per patient (17.4%). This estimate accounts solely for acquisition costs and does not include additional downstream resource implications, including nursing time required for administration, wastage of prepared infusions, the requirement for central intravenous access, or potential costs associated with line-related complications such as infection or thrombosis. Furthermore, it does not consider the impact on broader environmental costs associated with drug manufacturing, transport, and incineration of drug waste.

Adherence to the revised threshold was incomplete: supplementation was initiated below 3.6 mmol/L in 61.9% of post-guideline episodes, compared with 95.7% initiated below the former 4.5 mmol/L threshold pre-guideline. This comparison was not formally tested statistically, as the appropriateness criterion does not fully account for individualised clinical decision-making, which may have appropriately influenced deviation from guideline-defined prescribing criteria. For example, recent randomised evidence indicates that actively raising plasma potassium to a high-normal target (4.5-5.0 mmol/L) reduces the risk of ventricular arrhythmia, arrhythmia- or heart failure-related hospitalisation, and death in patients with an implantable cardioverter-defibrillator and severe left ventricular impairment, providing a plausible clinical rationale for deviating from a restrictive threshold in this specific subgroup [10].

Most importantly, no evidence of increased arrhythmia burden associated with the restrictive potassium threshold was identified. In neither audit period was a consistent increase in arrhythmia prevalence observed following supplementation events, and the proportion of patients experiencing any arrhythmia was 11/22 pre-guideline and 8/26 post guideline. The substantially higher total arrhythmia burden in the pre-guideline period, predominantly driven by atrial fibrillation (1301.5 vs 25.0 atrial fibrillation hours), is most plausibly explained by case-mix differences, specifically the admission of a higher proportion of patients with established or new-onset atrial fibrillation in the pre-guideline week, rather than any relationship with the supplementation threshold.

These findings align with prior evidence that guideline-driven restriction of IV potassium use is achievable without compromising patient outcomes. Min et al. reported a reduction in median IV potassium dose per patient from 215 to 80 mmol following the introduction of restrictive guidelines in a mixed ICU, while maintaining acceptable potassium homeostasis and without a significant increase in hypokalaemia duration (18.2% pre-guidelines vs 14.8% post-guidelines) [7]. Hoekstra et al. similarly demonstrated that a nurse-centred computerised potassium protocol compared to physician-driven regulation reduced the incidence of hypokalaemia and hyperkalaemia in surgical and cardiothoracic ICU patients [11]. The magnitude of reduction in the present study is consistent with these findings and likely reflects both the steeper threshold change and the lower baseline atrial fibrillation burden in the general ICU population studied.

This study has several limitations. As a single-centre quality improvement audit, the findings are inherently subject to residual confounding and limited external generalisability; ICUs with different patient populations, staffing models, prescribing cultures, or electronic record systems may not see comparable results, and compliance with the revised threshold depended on clinician adherence rather than mandatory prescribing controls. The short duration of data collection increases susceptibility to temporal variation in case-mix and clinical activity and may not fully reflect routine practice across longer timeframes. Although baseline demographic and clinical characteristics were broadly similar between groups, the relatively small sample size limits statistical power and increases the risk of type II error, particularly for clinically relevant outcomes such as arrhythmia incidence. Rhythm data were derived from continuous monitoring uploaded automatically to Epic but were not prospectively adjudicated against ECG or Holter-equivalent review; brief, self-terminating, or artefactual rhythms may therefore have been misclassified or missed, although this limitation applies equally to both audit periods and is unlikely to introduce differential bias. This study also does not include the use of other forms of IV supplementation, e.g., enteral potassium supplements, which may impact the need for IV supplementation. ICU length of stay was recorded but varied widely, from same-day admissions to prolonged stays, and was longer in the pre-guideline group (9.1 versus 6.3 days). Administrations and cost were therefore not indexed to ICU bed-days, and the 17.4% reduction in administrations per patient and the associated cost estimate may partly reflect shorter stays; indexing to ICU days will be included in follow-up audits. Measures that do not depend on length of stay, namely the proportion of episodes initiated at K⁺ ≥4.0 mmol/L and the lower pre-supplementation potassium, are therefore the more robust evidence of changed prescribing.

The study was also not powered for arrhythmia or other safety outcomes; it could not be stratified by ICU sub-specialty, and a before-and-after design cannot establish causation. Finally, lack of formal patient-level matching and adjustment for illness severity further limits causal inference regarding the observed effects. Longer-term, multi-centre evaluations with larger, risk-adjusted cohorts and adjudicated rhythm monitoring would be required to confirm the reproducibility and generalisability of these findings, ideally using a multi-centre interrupted time-series design over six to 12 months, stratification by ICU sub-specialty, computerised decision support to support adherence, and micro-costing and life cycle assessment to capture nursing time, central line use, waste and environmental impact.

## Conclusions

Implementation of a TIGHT-K-aligned restrictive intravenous potassium supplementation threshold in a general mixed adult ICU was associated with a substantial reduction in potassium administration with no signal of increased arrhythmia burden. These results potentially extend the applicability of restrictive supplementation strategies beyond cardiothoracic populations and suggest potential safety and resource benefits in broader intensive care practice. Larger, prospective multicentre studies with risk-adjusted analyses and standardised rhythm adjudication are required to confirm these findings and define their wider clinical and economic impact.
